# New Insights from Sum Frequency Generation Vibrational Spectroscopy into the Interactions of Islet Amyloid Polypeptides with Lipid Membranes

**DOI:** 10.1155/2016/7293063

**Published:** 2015-11-30

**Authors:** Li Fu, Zhuguang Wang, Victor S. Batista, Elsa C. Y. Yan

**Affiliations:** ^1^William R. Wiley Environmental Molecular Sciences Laboratory, Pacific Northwest National Laboratory, P.O. Box 999, Richland, WA 99352, USA; ^2^Department of Chemistry, Yale University, 225 Prospect Street, New Haven, CT 06520, USA

## Abstract

Studies of amyloid polypeptides on membrane surfaces have gained increasing attention in recent years. Several studies have revealed that membranes can catalyze protein aggregation and that the early products of amyloid aggregation can disrupt membrane integrity, increasing water permeability and inducing ion cytotoxicity. Nonetheless, probing aggregation of amyloid proteins on membrane surfaces is challenging. Surface-specific methods are required to discriminate contributions of aggregates at the membrane interface from those in the bulk phase and to characterize protein secondary structures *in situ* and in real time without the use of perturbing spectroscopic labels. Here, we review the most recent applications of sum frequency generation (SFG) vibrational spectroscopy applied in conjunction with computational modeling techniques, a joint experimental and computational methodology that has provided valuable insights into the aggregation of islet amyloid polypeptide (IAPP) on membrane surfaces. These applications show that SFG can provide detailed information about structures, kinetics, and orientation of IAPP during interfacial aggregation, relevant to the molecular mechanisms of type II diabetes. These recent advances demonstrate the promise of SFG as a new approach for studying amyloid diseases at the molecular level and for the rational drug design targeting early aggregation products on membrane surfaces.

## 1. Introduction

Amyloid aggregates formed by misfolded intrinsically disordered proteins are implicated in many diseases [1]. Here, we focus on human islet amyloid polypeptides (hIAPPs) that aggregate into parallel *β*-sheets upon the interaction with membrane surfaces [2–4]. The resulting aggregates are detrimental to pancreas *β*-cells, leading to the onset of type II diabetes [5–7], since they disrupt the membrane integrity [8–13], increasing the membrane permeability to water and ions [14]. Understanding surface-specific biomolecular interactions between hIAPP aggregates and lipid membranes at the molecular level is therefore crucial for revealing the molecular factors controlling amyloidogenesis.

Previous studies have relied mainly on bulk detection techniques, including circular dichroism (CD) [15], NMR [16–18], EPR [19, 20], 2D-IR [21–23], AIR-FTIR [24–26], fluorescence spectroscopy [27, 28], Raman spectroscopy [29], and infrared reflection absorption spectroscopy (IRRAS) [30, 31]. However, many specific questions concerning the interfacial region of interactions between membranes and proteins remain unclear. Some of the outstanding questions are as follows: Do the amyloid aggregates start forming at membrane surfaces, or in the bulk solution? How do the aggregates orient on membrane surfaces? Are the aggregation products parallel or antiparallel *β*-sheets? Does the orientation of the aggregate affect the integrity of cell membranes? What is the kinetics of misfolding at membrane surfaces? Is it different from misfolding in the bulk solution? Do molecular inhibitors affect aggregation on membrane surfaces? Recent developments in nonlinear sum frequency generation (SFG) vibrational spectroscopy have demonstrated SFG as an intrinsically surface-selective technique with submonolayer sensitivity and label-free detection capability, thus showing great promise to address the above questions, shedding light on the role of the membrane during the aggregation of hIAPP and other amyloid proteins [32, 33].

During the last three decades, we have witnessed the emergence and fast development of nonlinear optical spectroscopic techniques, among which SFG vibrational spectroscopy has gained tremendous attention. Unique advantages of SFG include surface selectivity, submonolayer sensitivity, chiral-selectivity, phase-sensitivity, and label-free detection capabilities [34–36], which make SFG a promising tool for structural characterization of interfaces, including a wide range of applications in material science [37], characterization of polymers [38, 39], and catalytic systems [40, 41]. Recently, applications have been extended to environmental [42–44] and biological systems [32, 33, 45–52] with unprecedented discoveries beyond the capabilities of conventional tools. For example, SFG has been used to study a variety of atmospherically significant systems at the vapor/aqueous interface to elucidate the organization and reactions in aerosols that contain inorganic/organic compounds [42–44], small molecules [42], and fatty acids [42] as solutes. Furthermore, SFG has been applied to probe the interfacial structures, orientation, and kinetics of biologically relevant molecules at interfaces, such as proteins [32, 33, 45–47], DNAs [48, 49], and lipids [45, 50–52], providing insights into the functions of these molecules at biological interfaces and facilitating further research into biomedical science and engineering. Nowadays, SFG is established as a valuable technique to understand physical, chemical, and biological processes at the molecular level [53]. In particular, applications to interfacial biological systems span across important research topics in membrane biophysics [54], surface self-assembly [55, 56], peptides at cell surfaces [57, 58], DNA hybridization [48, 59] and adsorption [60], and amyloid interactions with membrane surfaces [61–63].

This review focuses on the recent development and application of SFG for probing hIAPP interacting with lipid membranes and discusses the implications of hIAPP/membrane interactions in the studies of type II diabetes [14, 61, 62, 64, 65]. The review also summarizes the basic theoretical background and experimental methods of SFG, supplemented with a brief discussion about the application of SFG to other amyloidogenic proteins, and concludes with an outlook of SFG in applications to systems of interest in biological and medical sciences.

## 2. The SFG Method

### 2.1. Basic Principles of SFG

**Scheme 1:**
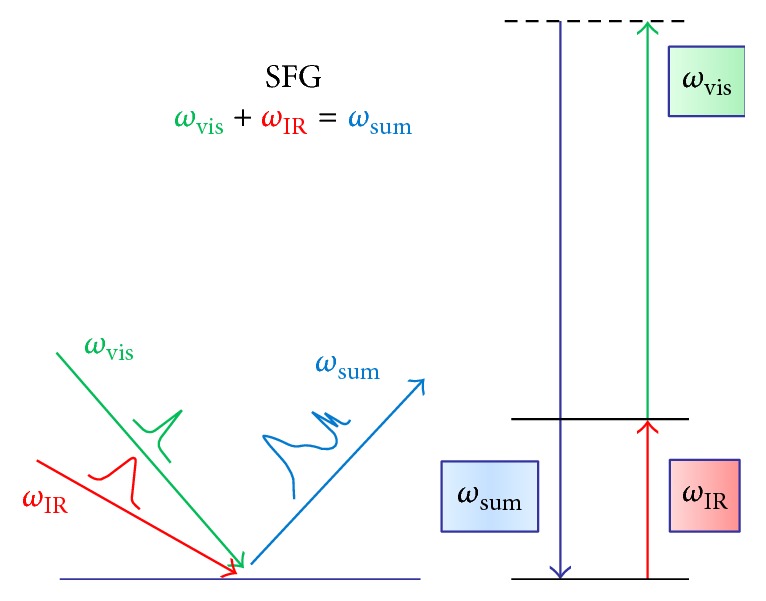
The second-order optical process of sum frequency generation vibrational spectroscopy.

Sum frequency generation (SFG) vibrational spectroscopy applies two laser beams that interact with the system of interest simultaneously [34–36, 66–69], one with visible (e.g., 532 nm) or near-infrared (e.g., 800 nm) frequency *ω*_vis_ and the other with infrared (IR) frequency *ω*_IR_ (Scheme 1). When the visible and infrared pulses overlap in time and space, light with the sum of the two frequencies *ω*_SFG_ = *ω*_vis_ + *ω*_IR_ is generated by the interaction of the incident beams with the system at the interface. Fixing *ω*_vis_ and scanning or dispersing IR frequencies *ω*_IR_ over a range, the SFG spectra are recorded by monitoring the SFG intensity as a function of *ω*_SFG_ with a monochromator and a CCD. The SFG signal is dramatically enhanced when *ω*_IR_ is in resonance with a vibrational frequency of a molecule at an interface, thus showing a peak in the spectrum. The SFG peaks typically exhibit homogeneous and inhomogeneous broadening, leading to various line-shapes due to the constructive or destructive interference with neighboring bands modulated by changes in the molecular orientation as induced by interactions with the surface and the surrounding environment [70]. Thus, the SFG spectra contain detailed structural information reported in terms of line-shape, peak position, and polarization dependence. Extracting that structural information from the SFG spectra, however, requires rigorous theoretical modeling and computer simulations. Hence, the combination of SFG and computational modeling can be used as a label-free and* in situ* analytical methodology for effective characterization of systems at interfaces. In the following sections, we will illustrate the applications of SFG to the studies of IAPP at membrane surfaces [14, 61, 62, 64, 65].

### 2.2. Surface-Specificity, Monolayer Sensitivity, and Polarization Dependence of SFG Spectroscopy

As a nonlinear optical technique, SFG measures the second-order susceptibility, *χ* ^(2)^, that gives intrinsic surface selectivity [33, 34, 71–74]. The second-order susceptibility, *χ* ^(2)^, is the direct product of the complex conjugate of the Raman polarizability derivative matrix (the same as the original matrix when derivative values are real numbers) and the IR dipole derivative vector and thus is a second-order tensor. The generated electric field of SFG signal is related to the electric field of the two incident laser beams, *E*_vis_ and *E*_IR_, via the tensor elements *χ*_*ijk*_^(2)^:

(1) $$\begin{array}{c} E_{\text{SFG}}^{i}\propto \underset{j,k}{\sum }\chi_{ijk}^{\left(2\right)}E_{\text{vis}}^{j}E_{\text{IR}}^{k}, \end{array}$$

where *i*, *j*, and *k* specify the direction of the Cartesian component of the optical fields and can be denoted by *x*, *y*, and *z*, in the laboratory coordinates.

The tensor elements of bulk phases with inversion symmetry (e.g., gas, solution, and amorphous solid) are isotropically averaged to zero, so long as the frequency of the visible beam is not in resonance with an electronic excitation [33, 73, 74]. This is because molecules rotate freely and diffuse, adopting random orientations. In contrast, molecules at surfaces give prominent SFG signals since they have ordered alignment across the surface region, and thereby their tensor elements are nonzero. Furthermore, the second-order susceptibility is proportional to the square of the molecular density at surfaces and thus is sensitive to the change of molecular coverage, enabling SFG to detect a monolayer of molecules at an interface. The monolayer sensitivity is critical for the characterization of biological samples that are difficult to purify in large quantities. As a result of such unique surface-specificity and monolayer sensitivity, the SFG method is free from contributions of the bulk medium and is thus an ideal optical method to probe membrane surfaces and their interactions with other biomolecules.

SFG spectroscopy has the intrinsic sensitivity to chiral structures because the measured second-order susceptibility tensor elements *χ*_*ijk*_^(2)^ are three-dimensional. When the three indices *i*, *j*, and *k* are distinct from one another (i.e., *i* ≠ *j* ≠ *k*), the susceptibility tensor captures the features of the chiral Cartesian coordinate system with three distinct axes and thus comprises information about the chirality at interfaces [33]. Therefore, surface chirality can be directly measured through *χ*_*ijk*  (*i*≠*j*≠*k*)_^(2)^ making chiral SFG spectroscopy highly sensitive and easier for interpretation, compared to more conventional chiroptical methods, such as circular dichroism, Raman optical activity, and optical rotation dispersion, which require higher-order couplings between electronic and magnetic dipoles. Due to its high chiral sensitivity, SFG could provide previously unattainable molecular information about protein and biomolecules in chiral supramolecular and hierarchic structures at surfaces [74, 75].

**Scheme 2:**
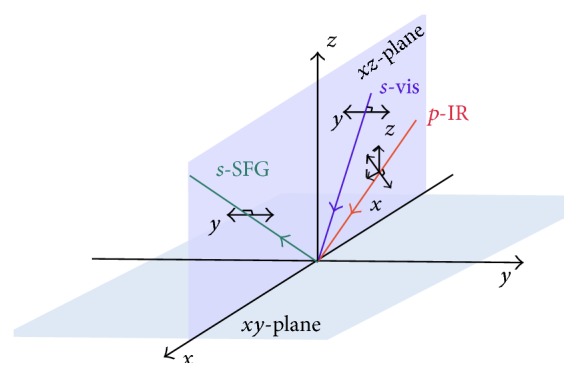
The *ssp* polarization setting in an SFG experiment: *s*-polarized SFG, *s*-polarized visible, and *p*-polarized IR beams. The projection electric field of *p*-polarized and *s*-polarized light onto the laboratory coordinates.

SFG measurements can be modulated by various polarization settings. For a particular experiment, one can modulate the incident visible and infrared beams and detect the SFG beam in either* s*- or* p*-polarization [66, 69], or even mixed polarizations [48, 72, 76]. With the use of only* s*- or* p*-polarization, there are in total 2 × 2 × 2 = 8 polarization settings for experimental geometries, including* ssp*,* ppp*,* sps*,* pss*,* spp*,* psp*,* pps*, and* sss*, where the first, second, and third indices indicate the polarization of the SFG, visible, and infrared beams, respectively (Scheme 2). The* psp*,* spp*, and* pps* polarization settings are chiral-selective and thus can be used to probe the chiral SFG spectra as discussed previously [33]. The others are achiral polarization settings that are sensitive to different vibrational modes. Altogether, chiral and achiral SFG spectroscopy can provide a comprehensive analysis of vibrational modes of chiral or nonchiral molecules at interfaces. In more advanced measurements, one can even determine the absolute orientation of molecules at interfaces by performing a global analysis of various polarization-modulated spectra [77]. With these capabilities, SFG can report on structures and orientations of molecules and proteins at surfaces, offering a methodology to address mechanistic questions on amyloid aggregation that would otherwise be difficult to tackle by using more conventional methods.

### 2.3. SFG Experiments

The setup of the SFG spectrometer has been extensively described [77–79]. Currently, scanning and broad-bandwidth SFG spectrometers are most commonly used. Scanning SFG spectrometers use picosecond pulsed IR and visible beams and scan the IR frequency stepwise at a fixed visible frequency [80]. A typical broad-bandwidth SFG spectrometer consists of a femtosecond (~100 fs) pulsed IR beam and a picosecond (2–100 ps) pulsed visible beam [81]. Because the femtosecond IR pulse includes a wide frequency range of more than 200 cm^−1^ in the mid-IR region, broad-bandwidth SFG spectrometers can acquire spectra by one shot without scanning the IR frequency. The one-shot scheme allows for monitoring the kinetics of protein conformational changes at interfaces [55, 82]. In this review, we focus on recent studies of hIAPP aggregates at membrane surfaces based on broad bandwidth SFG spectroscopy [14, 61, 62, 64, 65].

Chiral and achiral SFG spectra can be selectively collected using different polarization settings. Typically, one can use* psp*,* spp*, and* pps* to probe the chiral SFG spectra and* ssp*,* sps*, and* ppp* to probe the achiral SFG spectra [33]. When performing the chiral and achiral SFG measurements, one can control the polarization of the beams using appropriate wave-plates and polarizers. Chiral SFG is particularly useful for probing biomolecules because most secondary structures are chiral, such as *α*-helices and *β*-sheets. These chiral macromolecular structures are expected to show a strong chiroptical response in chiral SFG while the solvent and other molecules lacking macroscopic chiral structures are silent in the chiral SFG spectra. Thus, chiral SFG spectra of biomacromolecules are less affected or distorted by signals from solvent or other achiral molecules, simplifying the spectral analysis and interpretation.

**Scheme 3:**
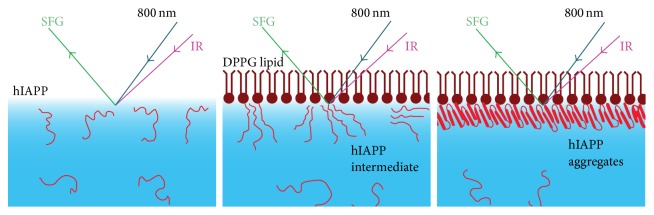
Illustration of adsorption of hIAPP on a lipid monolayer and the SFG experiment for probing the hIAPP aggregations at the lipid/water interface. Adapted from [62] with permission. Copyright 2010 American Chemical Society.

The choice of surface platform is another important factor for efficiently probing biomolecules at interfaces. For studies of hIAPP, there are two applicable surface platforms that allow for probing the lipid/aqueous interfacial region [83]. One surface platform is made by spreading a lipid monolayer onto the water surface [31], while the other is made by fabricating a supported lipid bilayer on a solid substrate, using the Langmuir-Blodgett (LB)/Langmuir-Schaefer (LS) method [51]. Both platforms have been widely used as mimics of biomembranes. Here, we will focus on the hIAPP studies that have been carried out using the platform of lipid monolayer. Briefly, the experimental procedure (Scheme 3) [62] starts with adding hIAPP to an aqueous buffer and then spreading the lipid monolayer at the air/water interface. The hIAPP will adsorb at the interface and interact with the lipid membrane. SFG spectroscopy can then be used to monitor lipid-induced conformational changes in hIAPP* in situ* and in real time at the interface.

## 3. SFG Probes the Early Stages of hIAPP Aggregation at Membrane Interfaces

The early stages of hIAPP aggregation at interfaces involve hIAPP-membrane interactions associated with the pathogenic mechanism of type II diabetes [6, 84, 85]. However, it has been challenging to probe how hIAPP adsorbs onto the interface and whether hIAPP undergoes structural and orientation changes that might induce toxicity to pancreatic *β*-cells. Previous studies of the early stages of hIAPP aggregation mainly focused on bulk detection using methods such as CD, NMR, 2D-IR, and fluorescence spectroscopy that are not surface-sensitive or require spectroscopic labeling [9].

To overcome these challenges, Winter and coworkers applied infrared reflection absorption spectroscopy (IRRAS) and found *β*-sheets structures formed in the process of hIAPP aggregation at the air/water interface with a negatively charged lipid [31]. Inspired by the earlier IRRAS work, our group used SFG spectroscopy to probe hIAPP aggregation at membrane surfaces* in situ* and in real time monitoring the amide I and N-H stretching vibrational modes [61, 62]. Protein structures, including *α*-helices and *β*-sheets, are formed by hydrogen bonding interactions between the amide and N-H groups along the protein backbone. Thus, the vibrational frequency and line-shape of the amide I and N-H stretching modes are sensitive markers for distinguishing protein secondary structures [86, 87]. In conventional vibrational studies, the O-H bending and O-H stretching of water overlap with the amide I and N-H stretching modes, masking the characteristic bands of secondary structures, the peak assignment, and characterization of the protein secondary structure. In contrast, chiral SFG provides high-quality vibrational spectra revealing conformational changes previously undetectable by using conventional methods, since it probes only the interfacial molecules without any significant vibrational background from water solvent. Below is a summary of such vibrational SFG studies in the amide I and N-H stretching regions probing the early aggregation of hIAPP upon the interaction with membrane surfaces.

### 3.1. hIAPP Aggregation at Interfaces Probed by Amide I SFG Signals

**Figure 1:**
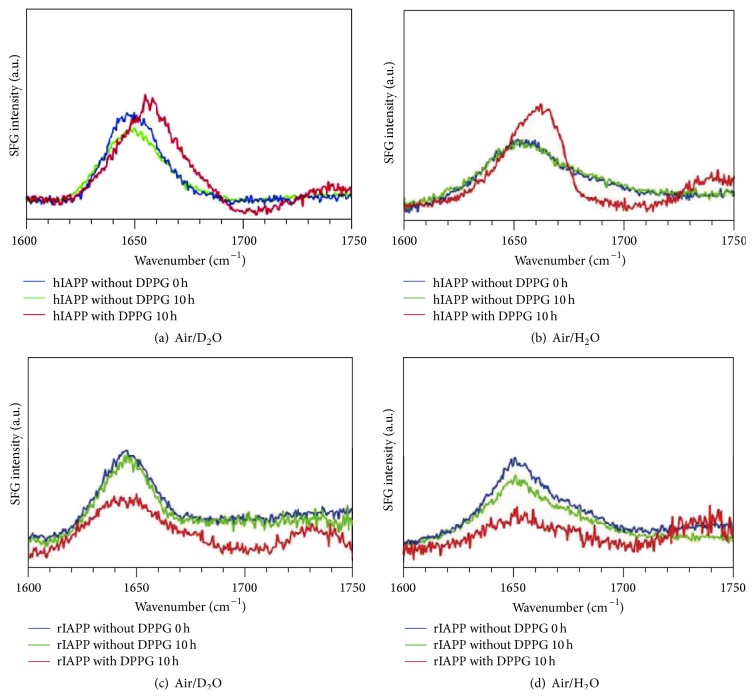
The* ssp* (achiral) SFG spectra of IAPPs. Human IAPP without DPPG (*t* = 0 h and *t* = 10 h) and with DPPG at *t* = 10 h at the (a) air/D_2_O and (b) air/H_2_O interfaces; rat IAPP without DPPG (*t* = 0 h and *t* = 10 h) and with DPPG at *t* = 10 h at the (c) air/D_2_O and (d) air/H_2_O interfaces. Adapted from [62] with permission. Copyright 2010 American Chemical Society.

First, our group has focused on experiments for both human IAPP (hIAPP) and rat IAPP (rIAPP) that probed the amide I region in both achiral and chiral SFG spectra [32, 33, 61, 62, 65, 88]. The two peptides are different by only six amino acids. Remarkably, the rIAPP does not aggregate into amyloids, making it an ideal control system for SFG studies. The achiral SFG spectra (Figure 1) were collected in the absence and presence of the negatively charged lipid dipalmitoylphosphoglycerol (DPPG). In the absence of DPPG, both hIAPP and rIAPP show amide I peaks at 1650 cm^−1^, suggesting the presence of both peptides at the air/water interface. Moreover, the spectra do not change over 10 hours, suggesting no noticeable structural changes. In contrast, with a DPPG monolayer and after incubation for 10 hours, the amide I band of hIAPP changes dramatically in terms of both the peak position and the line-shape. In contrast, the spectra of rIAPP remained unchanged. Since the frequency of the amide I band varies with protein secondary structures, the different spectroscopic responses from hIAPP and rIAPP indicate that hIAPP exhibits structural changes upon interaction with a DPPG monolayer, while rIAPP does not. Figure 1 shows similar results for experiments in H_2_O and D_2_O, suggesting that isotopic effects are negligible on hIAPP aggregation.

A closer look at the spectral change of hIAPP incubated with DPPG after 10 hours shows that the amide I peak position is blue-shifted by 10 cm^−1^, from ~1650 to ~1660 cm^−1^, and there is an additional peak at 1750 cm^−1^ corresponding to the carbonyl stretch of the DPPG lipid [87]. Nonetheless, it is still challenging to specify what structural changes are involved at the lipid/aqueous interface. To address this question, we applied chiral SFG.

**Figure 2:**
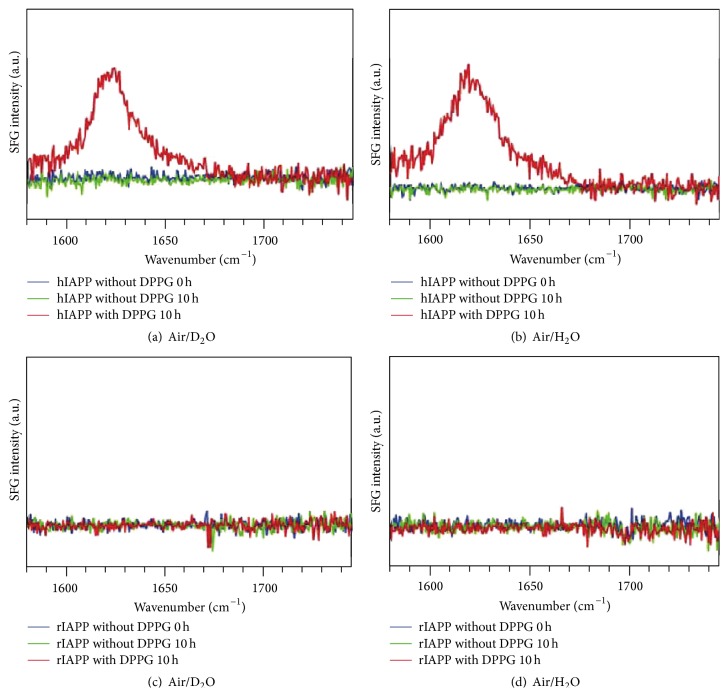
The* psp* (chiral) SFG spectra of IAPPs. Human IAPP without DPPG (*t* = 0 h and *t* = 10 h) and with DPPG at *t* = 10 h at the (a) air/D_2_O and (b) air/H_2_O interfaces; rat IAPP without DPPG (*t* = 0 h and *t* = 10 h) and with DPPG at *t* = 10 h at the (c) air/D_2_O and (d) air/H_2_O interfaces. Adapted from [62] with permission. Copyright 2010 American Chemical Society.

The chiral SFG measurements (Figure 2) show more interesting phenomena. Without the DPPG lipid, neither hIAPP nor rIAPP shows detectable chiral SFG signal in the amide I region. The lack of signal is not surprising since the native structures of hIAPP and rIAPP are disordered and do not adopt any chiral conformation. However, after incubating with DPPG for 10 hours, hIAPP shows a strong chiral SFG signal at 1622 cm^−1^ with a shoulder at 1660 cm^−1^. The low-frequency amide I band at 1622 cm^−1^ and a shoulder-peak at 1660 cm^−1^ are characteristic of parallel *β*-sheets [86, 87]. Thus, these results indicate that hIAPP forms parallel *β*-sheets upon the interaction with DPPG at the lipid/water interface.

Altogether, the achiral and chiral SFG studies of hIAPP in the amide I region demonstrate that both hIAPP and rIAPP can adsorb onto the air/water interface. Furthermore, hIAPP undergoes structural changes from disordered structures to parallel *β*-sheets upon the interaction with the surface of a lipid monolayer. Moreover, as a control system, rIAPP remains unchanged with and without lipid at the surface, revealing clear differences in the behavior of hIAPP and rIAPP at the lipid/aqueous interface. Detailed analyses of these spectral data have provided structural, kinetic, and orientation information, discussed in the following sections.

### 3.2. SFG Allows for Kinetic Studies of hIAPP Misfolding at the Early Stage

**Figure 3:**
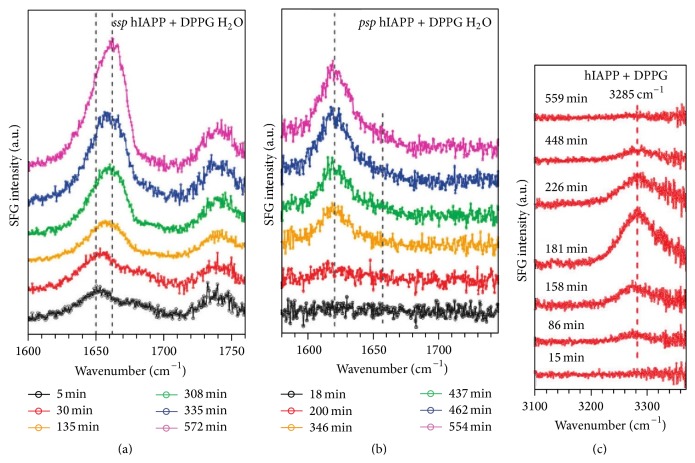
Kinetics of human IAPP aggregates at the lipid/H_2_O interface probed by time-dependent SFG spectra in the amide I region using (a)* ssp* (achiral) and (b)* psp* (chiral) polarization in the presence of DPPG and (c) in the N-H stretching region using* psp* (chiral) polarization in the presence of DPPG. Adapted from [61, 62] with permission. Copyright 2010 and 2011 American Chemical Society.

The chiral and achiral SFG spectra have been collected over time (Figure 3) [61, 62] to explore the kinetics of aggregation of hIAPP at the interface.  Figure 3(a) shows that during the aggregation process the achiral amide I band of hIAPP gradually shifts to higher frequency with increased intensity, suggesting conformational changes in hIAPP. The increase in intensity reflects more ordered structures of hIAPP aggregates because SFG signals are sensitive to the ordering of the molecules at interfaces.  Figure 3(b) shows that the chiral amide I signal at 1622 cm^−1^ starts emerging after three hours and keeps increasing with an appearance of a shoulder-peak at 1660 cm^−1^. This result not only suggests the formation of parallel *β*-sheets but also confirms the ordering of hIAPP during the aggregation process.

**Figure 4:**
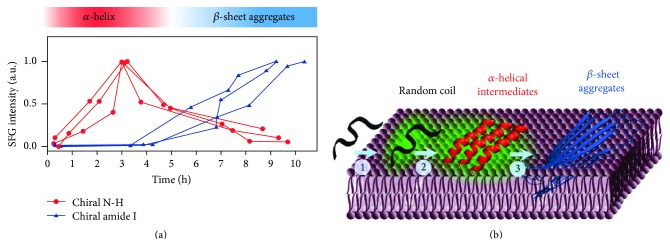
The misfolding pathway of hIAPP on membrane surfaces. (a) Chiral SFG intensities of the chiral N-H stretching (3280 cm^−1^) and amide I signals (1620 cm^−1^) as a function of time from triplicate experiments. (b) The model mechanism on hIAPP aggregation at the membrane surface: adsorption of disordered hIAPP onto membrane leads to formation of *α*-helical intermediates which are then converted to *β*-sheet aggregates. Adapted from [61] with permission. Copyright 2011 American Chemical Society.

The successful use of the amide I band to probe the kinetics of *β*-sheet formation in hIAPP inspired us to use SFG signals from the peptide backbone in other vibrational regions, such as N-H stretching (amide A) [61]. We probed the chiral N-H stretching signals of hIAPP during the aggregation process of hIAPP under the same experimental conditions and observed unique spectral features. The chiral SFG spectra in Figure 3(c) show that initially there are no N-H stretching signals, but a peak at 3280 cm^−1^ gradually builds up and reaches a maximum value after roughly three hours of interaction with DPPG and then slowly vanishes after 10 hours. This transient chiral N-H stretching signal clearly reveals an intermediate in the hIAPP aggregation process. To investigate further the structure of this intermediate, we obtained the chiral N-H stretching spectra of several model proteins in *α*-helical structures. We concluded that the chiral SFG N-H stretching mode at 3280 cm^−1^ is due to *α*-helical structures [61]. A combination of the results of kinetic studies using the amide I (Figure 3(b)) and N-H stretching bands (Figure 3(c)) reveals an important finding: the appearance of the N-H stretching peak reaches a maximum and starts to disappear prior to the accumulation of the chiral amide I signal (Figure 4(a)), providing a molecular picture of hIAPP misfolding at membrane surfaces, where hIAPP initially adsorbs to the membrane surface as a random coil and then forms *α*-helical intermediates, which subsequently convert into parallel *β*-sheet aggregates.

As the first kinetic study using chiral and achiral SFG to probe conformational changes of proteins at interfaces* in situ* and in real time, the above studies demonstrate SFG as a method of high selectivity and sensitivity not only for characterizing protein secondary structures but also for studying the kinetics of conformational changes at interfaces. The N-H stretching and amide I bands are two well-separated vibrational regions that can be used synergistically for revealing aspects of the molecular mechanism of aggregation at interfaces. The kinetic data obtained at the lipid/water interface by SFG spectroscopy can be compared to measurements in the bulk solution based on conventional physical methods, yielding a better understanding of the role that the membrane surface plays in the amyloid aggregation process. This methodology is expected to find applications in testing the efficacy of drug candidates that inhibit the aggregation of hIAPP at lipid/water interfaces, as illustrated in the following section.

## 4. Inhibition of the hIAPP Aggregation at Membrane Surface

Several studies have shown that the aggregation of hIAPP is associated with the disruption of membrane integrity and death of pancreas cells [9, 11, 12]. Hence, the search for inhibitors of hIAPP aggregate formation has been a strategy explored for drug development [77, 89]. Previously, most of the screening of drug candidates inhibiting hIAPP aggregation has been tested in the bulk aqueous solution. However, inhibitors that work in the bulk may have low efficacy on membrane surfaces. Bonn and coworkers applied SFG to address this issue [64] and have confirmed that the surface indeed plays an important role in reducing the inhibition of hIAPP aggregation by drug candidates.

### 4.1. EGCG Shows Less Inhibition of hIAPP Fibril Formation at Interfaces

Bonn and coworkers studied (−)-epigallocatechin gallate (EGCG) as an inhibitor for hIAPP aggregation and compared its effects in the bulk solution and on membrane surfaces [64]. EGCG is a natural product found in green tea and belongs to a class of inhibitors containing polyphenols. Previous studies showed that EGCG can effectively inhibit the misfolding and fibrillation of hIAPP and even disaggregate *β*-sheet-rich amyloids in the bulk solution [90, 91]. However, the inhibitive effect of hIAPP at the interface remains unclear.

**Figure 5:**
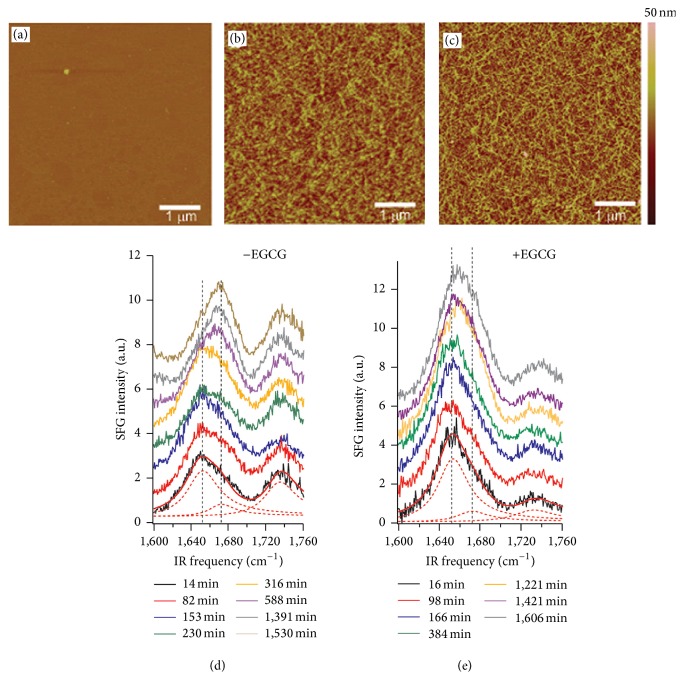
Kinetics study using AFM and SFG measurements during hIAPP aggregation at the phospholipid interface in the presence, or absence, of EGCG. AFM images of hIAPP with lipid for (a) 10 and (b) 1020 min in the absence of EGCG and (c) AFM image after hIAPP aggregation in the presence of EGCG for 1020 min. SFG spectra of hIAPP with phospholipid in the amid I region (d) in the absence of, and (e) in the presence of, EGCG. Adapted from [64] with permission. Copyright 2012 American Chemical Society.

Bonn and coworkers combined achiral SFG spectroscopy with thioflavin T (ThT) fluorescence and atomic force microscopy (AFM) for bulk analysis to examine the effect of EGCG on inhibiting hIAPP aggregation at the lipid/water interface [64]. They used the amide I band of hIAPP in the achiral SFG spectra to monitor the kinetics of the formation of *β*-sheet fibrils. In the absence of EGCG at the lipid/water interface, the time-dependent SFG spectra (Figure 5(d)) show blue-shifts in peak position and increasing intensities of amide I band, similar to the observations made by Fu et al. (Figure 3(a)). In the presence of EGCG, blue-shifts in peak positions can still be observed (Figure 5(e)), indicating the formation of *β*-sheets. This is confirmed by the AFM images of the sample at interfaces transferred onto mica. The AFM images show the formation of fibrils on the film made from hIAPP at the air/water interface with lipid in the presence of EGCG after incubating for ~17 hours (Figure 5(c)). The extent of fibril formation is similar to that in the absence of EGCG (Figure 5(b)). The results at the interface elicit the hypothesis that EGCG has a reduced inhibitive effect on hIAPP aggregation at the lipid/water interface.

**Figure 6:**
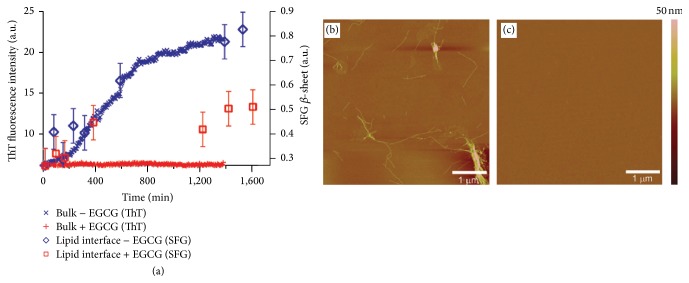
Bonn and coworkers compared the inhibitory effect of EGCG on hIAPP fibrillation in the bulk and at the phospholipid interface. (a) In the bulk, the formation of hIAPP amyloid fibrils was measured using a ThT fluorescence assay. The sigmoidal increase in fluorescence signal in the absence of EGCG (×) indicates the formation of hIAPP fibrils, and the low fluorescence signal in the presence of EGCG at a 1 : 1 molar ratio (+) suggests the inhibition of fibril formation. At the phospholipid interface, in absence (⋄) and presence (□) of EGCG, SFG spectra measure the formation of *β*-sheets. (b) AFM also shows hIAPP fibrils formed in the absence of EGCG and (c) EGCG completely inhibits fibril formation of hIAPP. Adapted from [64] with permission. Copyright 2012 American Chemical Society.

To test this hypothesis, a comparison is made for the inhibitive effect of EGCG on hIAPP aggregation at the interface versus in the bulk. The amount of *β*-sheets formed at the lipid/water interface in both the presence and the absence of EGCG is quantitatively estimated by the deconvolution of each time-dependent spectrum using the Lorentzian line-shape fitting. The time-dependent *β*-sheet component deconvoluted from the SFG spectra is plotted in Figure 6(a) (⋄ and □ curves), along with the time-dependent fluorescence intensity from hIAPP aggregates in the bulk (× and + curves). The flat + curve in Figure 6(a) suggests a lack of amyloid fibril formation in the bulk in the presence of EGCG, which is further confirmed by the AFM image in the presence (Figure 6(c)) of EGCG. Therefore, both the fluorescence and the AFM results indicate a strong inhibition of EGCG on hIAPP aggregation in the bulk. On the other hand, the *β*-sheet component deconvoluted from the SFG spectra is still increasing with time at the lipid/water interface in the presence of EGCG (Figure 6(a), □ curve). The above comparison demonstrates that the inhibitive effect of EGCG on hIAPP is indeed reduced at the interface.

### 4.2. Consideration of Membrane Effects for Drug Design

The reduced efficacy of EGCG as an inhibitor at membrane surfaces may be due to the role of the surface in controlling the structure, orientation, and dynamics of the aggregation process. The proposed inhibitory mechanism for EGCG involves the binding of the phenol groups to the hydrophobic aromatic side chains of hIAPP. In the bulk, both the inhibitor and the hIAPP diffuse freely. Thus, the inhibitor may bind to the hIAPP aromatic groups more effectively. At the amphipathic membrane-water interface, however, hIAPP is anchored with a specific orientation leaving the hydrophobic *β*-strands pointing towards each other, buried inside the membrane phase, while the hydrophilic side chains make contact with water solvent and the lipid polar head groups. This specific orientation suppresses free diffusion of hIAPP and makes it difficult for EGCG to bind. On the other hand, the small EGCG molecules with multiple phenol groups are more soluble in the bulk, potentially leading to low surface population, which further reduce its efficacy.

The SFG study demonstrates that the effect of the surface can be a critical factor to be considered in the drug design for type II diabetes. The pathogenic origin of type II diabetes has been proposed to be linked to the hIAPP aggregation process and the disruption of membrane integrity [5]. Therefore, it is important to screen drug candidates that might affect aggregate/membrane interactions, by changing the hIAPP interfacial orientation, conformation, or dynamics. The resulting conformational changes could be probed by SFG as described in the following section focused on the orientation of aggregates at the lipid/water interface.

## 5. Orientation of hIAPP Aggregates at Lipid Membrane Surfaces

Understanding, at the molecular level, whether hIAPP aggregates disrupt cell membranes could provide valuable insights into the pathogenic mechanisms of type II diabetes. Disruption due to a specific orientation of the hIAPP aggregates adsorbed on the membrane surface might increase the membrane permeability to water and ions. However, determining the interfacial orientation of complex biomolecules by conventional methods is challenging. In particular, complex hIAPP aggregates in the form of pleated parallel *β*-sheets pose significant challenges for both theory and experiments [92–94]. We have combined molecular dynamics and divide-and-conquer* ab initio* quantum chemistry calculations of hyperpolarizability derivatives with respect to normal modes to simulate the chiral SFG spectroscopy of hIAPP [65]. Our simulations found that the hIAPP aggregates are neither parallel nor perpendicular to the membrane surface but rather inserted into the lipid membrane tilted at an angle of about 45° relative to the surface normal. The resulting orientation optimizes amphiphilic interactions by exposing hydrophilic domains of the aggregate to the aqueous phase and hydrophobic parts to the lipids. Such “detergent-like” orientation is expected to cause significant disruption of the cell membrane.

This section describes the theoretical and experimental analyses of chiral SFG spectra necessary to retrieve the orientation of the hIAPP aggregate at the interface.

**Figure 7:**
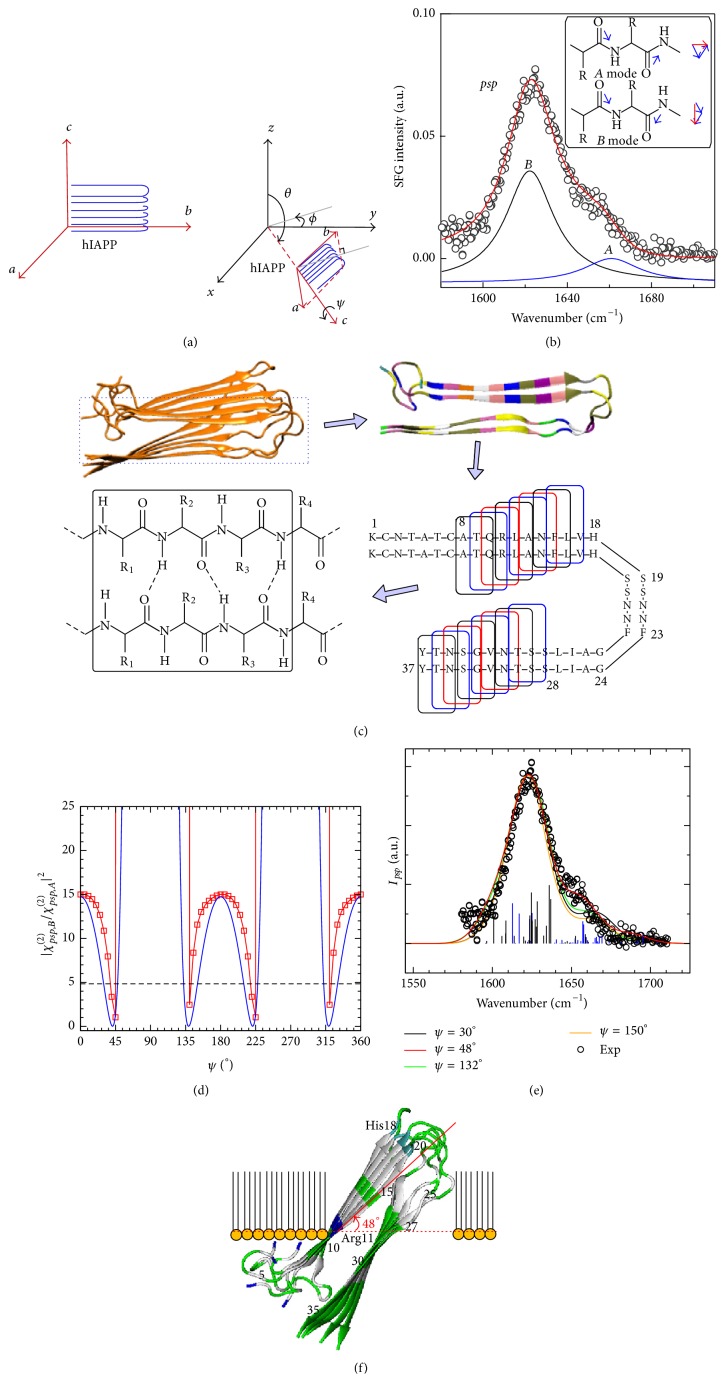
Determination of the orientation of human IAPP aggregates at the lipid/water interface. (a) Definition of three orientation angles (*ϕ*, *θ*, *ψ*) for Euler transformation from the laboratory to the molecular frame. (b)* psp* (chiral) SFG spectrum of hIAPP aggregates in the amide I region. *A* and *B* denote the characteristic peaks for amide I symmetric and antisymmetric modes. (c) Scheme showing the divide-and-conquer method for simulations of the SFG spectra from calculations of hyperpolarizability derivatives with respect to normal mode displacements in human IAPP aggregates. (d) Relationship between the intensity ratio of the *B* mode to the *A* mode and orientation angle *ψ*. The blue curve is obtained analytically from (1), and the red curve is obtained numerically. (e) Chiral SFG spectra of human IAPP aggregates simulated for various orientations at the interface. (f) Visualized orientation of the human IAPP aggregates at the lipid/aqueous interface. Adapted from [65] with permission. Copyright 2012 Elsevier.

The chiral SFG spectrum of the aggregated hIAPP at the membrane surface shows a dominant peak at 1620 cm^−1^ with a shoulder-peak at 1660 cm^−1^ in the amide I region (Figure 7(b)). These two peaks correspond to the antisymmetric (*B*-mode) and symmetric (*A*-mode) bands of parallel *β*-sheets. Detailed analyses of the molecular symmetry and vibrational coupling indicate that their relative ratio of intensities (*I*_*B*_/*I*_*A*_) is correlated with the orientation of the *β*-sheet

(2) IB/Aχpsp,B2χpsp,A22=tan2⁡ψβbca,Bβacb,A+1−tan2⁡ψβbac,Bβacb,A2,

where *ψ* is the angle between the *β*-strand and the interface, as defined in Figure 7(a). The hyperpolarizability elements (*β*_*acb*,*A*_, *β*_*bca*,*B*_, *β*_*bac*,*B*_) provide the specific molecular property of the hIAPP aggregates, with *a*, *b*, *c* referring to the Cartesian coordinates in the molecular frame of the hIAPP parallel *β*-sheet, where *b* is the direction parallel to the *β*-strand and *c* points to the axis of propagation of intermolecular *β*-sheets (Figure 7(a)). From (2), it is clear that the orientation angle *ψ* can be determined as the value that matches the experimental ratio of SFG intensities for the *B* and *A* bands (*I*_*B*/*A*_). The intensity ratio is measured to be 4.8 from the fitted amide I spectrum (Figure 7(b)). Consequently, knowing the values of three hyperpolarizability elements (*β*_*acb*,*A*_, *β*_*bca*,*B*_, *β*_*bac*,*B*_) in (2) allows the determination of the average orientation (*ψ*) determined by SFG spectroscopy.

**Figure 8:**
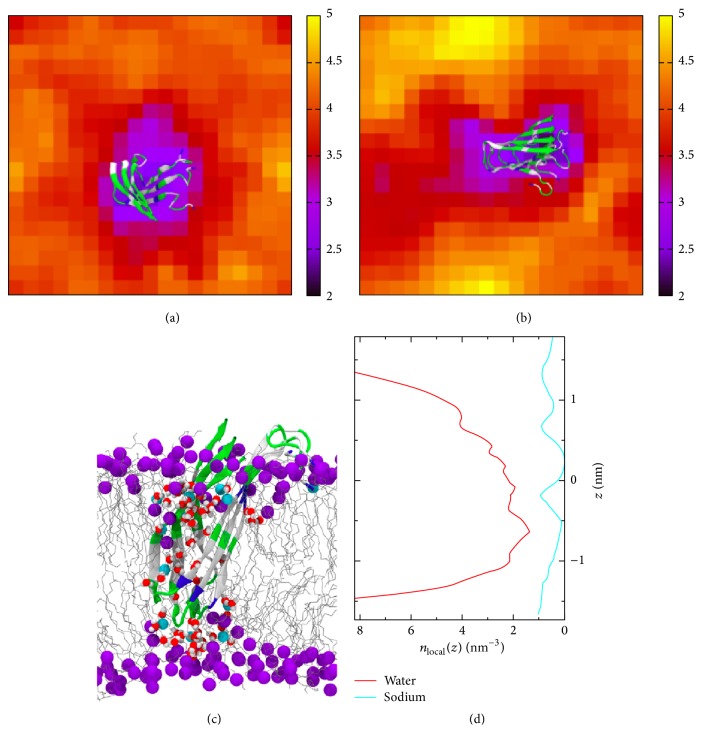
Bilayer thickness around embedded hIAPP for (a) trimer and (b) tetramer aggregates, as described by molecular dynamics simulations. Color key: bilayer thickness (in nm) is mapped to the corresponding colors. (c) Water channel formed by the hIAPP trimer in the DPPG bilayer, showing water molecules (red and white) and Na^+^ ions (blue vdW spheres) percolating through the bilayer. (d) Average particle density of water and Na^+^ ions within the membrane. Adapted from [14] with permission. Copyright 2013 by the Biophysical Society.

We have applied a divide-and-conquer approach that fragments the hIAPP aggregate into domains amenable to quantum chemistry calculations and computes the hyperpolarizability elements of the constituent fragments at the density functional theory (DFT) level. Specifically, the NMR structure [18] was divided into 16 tripeptide pairs in the *β*-sheet region, and the hyperpolarizability of each tripeptide pair was calculated by DFT (Figure 7(c)). The overall hyperpolarizability of hIAPP aggregates is then integrated from the hyperpolarizability elements of the individual tripeptide pairs. The plot of the intensity ratio of the amide I peaks as a function of the orientation of the parallel *β*-sheet shows that the orientation *ψ* = 45–48° has the best agreement with experimental data, suggesting that the hIAPP aggregates orient with the *β*-strand at *ψ* ≈ 45° from the surface. The tilted orientation of hIAPP *β*-sheet aggregates at the lipid/water interface suggests that significant disruption might be caused on the lipid membrane. These findings are supported by molecular dynamics simulations that analyzed the orientation and stability of hIAPP aggregates at DPPG/water interfaces [14]. The simulation shows that hIAPP gets inserted into lipid monolayers at about *ψ* = 40° and in lipid bilayers at a tilted angle of *ψ* = 60°, as shown in Figure 8, leading to water permeation and Na^+^ percolation through the membrane supporting the hypothesis of ion-cytotoxicity for islet *β*-cells [14].

These molecular dynamics simulations support the unique capabilities of chiral SFG spectroscopy for probing the orientation of *β*-sheet amyloid aggregates, providing fundamental insights that should be particularly relevant for understanding amyloid diseases at the molecular level. The combination of computational modeling and SFG spectroscopy thus provides a valuable methodology to identify potential noncompetitive inhibitors that might change the conformation and orientation of hIAPP aggregates and consequently reduce their toxicity as implicated in amyloid diseases.

## 6. Perspectives and Challenges of SFG in Biological and Medical Applications

In summary, we have reviewed the application of SFG spectroscopy to study the amyloidogenesis of hIAPP interacting with membrane surfaces [14, 61, 62, 64, 65]. We have shown that SFG can reveal structural and dynamic information characterizing the formation of aggregates* in situ* and in real time by using different polarization settings and probing in different vibrational regions. The versatility of SFG experiments also provides ample information that allows for elucidating the orientation of hIAPP aggregates at the water/lipid interface. With the capabilities of probing and characterizing structure, orientation, and dynamics, studies based on SFG spectroscopy can provide valuable insights into membrane/protein interactions that are critical to a wide range of pathological diseases, including amyloidogenesis and cytotoxicity to pancreas *β*-cells leading to the onset of type II diabetes. Based on these findings, potential drug candidates that specifically target the early aggregation of hIAPP at the membrane/water interface are currently being proposed and tested by using SFG spectroscopy to guide the rational design of drugs for treatment of type II diabetes.

Studies of other amyloid diseases such as Alzheimer's, Parkinson's, Huntington's, and Prion diseases could also benefit from SFG techniques. In fact, Luo and coworkers have already applied SFG to study the membrane-mediated structural change of prion protein fragments and characterized the concentration dependence of structures and orientation for prion oligomers [95]. In addition, Weidner and coworkers have performed SFG studies to investigate oligomerization of lysozyme at membrane surfaces, where they simultaneously monitored conformational states of lysozyme and the organization of lipid molecules in contact with aqueous buffer at various values of pH [63].

It is foreseeable that SFG spectroscopy can be extended to a wider range of studies critical for the mechanistic understanding of amyloid diseases and drug development. Potential applications include studies of the interactions of amyloid proteins with components in cell membranes (e.g., cholesterol, membrane protein, and glycolipid) [96]; other relevant biomolecules (e.g., insulin [97] and sphingolipid); and potential drug candidates (e.g., small aromatic organic molecule and peptide analogue of amyloid protein). Those applications could provide valuable insights into amyloidogenic intermediates during the onset of membrane diseases.

Given the potential applications of SFG in the investigation of amyloidogenesis, two major challenges remain in order to develop SFG into a general biophysical tool for biological and biomedical research. These challenges include the development of efficient methods for acquisition of high-quality spectroscopic data and unequivocal interpretation of the spectra. On one hand, experimentalists in the SFG field have been striving to improve the instrumentation for SFG spectroscopy to enhance the quality of the data. Broad bandwidth and even ultrabroad bandwidth SFG spectroscopy have been developed to cover a wider vibrational frequency range with one-shot measurement [56], shortening the time for spectral acquisition and rendering it possible to monitor kinetics of structural and orientational changes. High-resolution SFG spectroscopy can characterize surfaces and interfaces with unprecedented subwavenumber (<1 cm^−1^) resolution [98], providing detailed molecular information necessary to understand structure/function relations in physical and biological processes [99–101]. In addition, heterodyne-detected SFG spectroscopy has been developed to enhance the signal level of SFG spectra [102–107]. Moreover, 2-dimensional SFG spectroscopy and SFG microscopy have emerged as complementary techniques for the characterization of couplings and interactions in biomolecules [108–110]. On the other hand, theoretical methods for modeling SFG spectroscopy are critical for the interpretation of the SFG data. Methodologies that combine the essence of traditional theories of vibrational spectroscopy and the power of high-performance computing continue to be improved for more efficient calculations [111, 112] that provided rigorous first-principle interpretations of the experimental data in terms of structure/orientation relations of biological systems at interfaces. Efforts have been focused on accurate computations of molecular hyperpolarizabilities for different secondary structures of proteins, critical for extracting information about molecular orientation at membrane surfaces. These advancements made by strong collaborations between experimentalists and theoreticians in the field of SFG spectroscopy are expected to continue to produce valuable insights into a broad range of problems in chemical, biological, and biomedical sciences.

## References

[1] Dyson H. J., Wright P. E. (2005). Intrinsically unstructured proteins and their functions. *Nature Reviews Molecular Cell Biology*.

[2] Goldsbury C., Goldie K., Pellaud J. (2000). Amyloid fibril formation from full-length and fragments of amylin. *Journal of Structural Biology*.

[3] Kajava A. V., Aebi U., Steven A. C. (2005). The parallel superpleated beta-structure as a model for amyloid fibrils of human amylin. *Journal of Molecular Biology*.

[4] Knight J. D., Hebda J. A., Miranker A. D. (2006). Conserved and cooperative assembly of membrane-bound *α*-helical states of islet amyloid polypeptide. *Biochemistry*.

[5] Janson J., Ashley R. H., Harrison D., McIntyre S., Butler P. C. (1999). The mechanism of islet amyloid polypeptide toxicity is membrane disruption by intermediate-sized toxic amyloid particles. *Diabetes*.

[6] Höppener J. W. M., Ahrén B., Lips C. J. M. (2000). Islet amyloid and type 2 diabetes mellitus. *The New England Journal of Medicine*.

[7] Höppener J. W. M., Lips C. J. M. (2006). Role of islet amyloid in type 2 diabetes mellitus. *International Journal of Biochemistry and Cell Biology*.

[8] Jayasinghe S. A., Langen R. (2005). Lipid membranes modulate the structure of islet amyloid polypeptide. *Biochemistry*.

[9] Jayasinghe S. A., Langen R. (2007). Membrane interaction of islet amyloid polypeptide. *Biochimica et Biophysica Acta (BBA)—Biomembranes*.

[10] Hebda J. A., Miranker A. D. (2009). The interplay of catalysis and toxicity by amyloid intermediates on lipid bilayers: insights from type II diabetes. *Annual Review of Biophysics*.

[11] Engel M. F. M. (2009). Membrane permeabilization by Islet Amyloid Polypeptide. *Chemistry and Physics of Lipids*.

[12] Engel M. F. M., Khemtémourian L., Kleijer C. C. (2008). Membrane damage by human islet amyloid polypeptide through fibril growth at the membrane. *Proceedings of the National Academy of Sciences of the United States of America*.

[13] Knight J. D., Miranker A. D. (2004). Phospholipid catalysis of diabetic amyloid assembly. *Journal of Molecular Biology*.

[14] Poojari C., Xiao D., Batista V. S., Strodel B. (2013). Membrane permeation induced by aggregates of human islet amyloid polypeptides. *Biophysical Journal*.

[15] Kayed R., Bernhagen J., Greenfield N. (1999). Conformational transitions of islet amyloid polypeptide (IAPP) in amyloid formation in vitro. *Journal of Molecular Biology*.

[16] Nielsen J. T., Bjerring M., Jeppesen M. D. (2009). Unique identification of supramolecular structures in amyloid fibrils by solid-state NMR spectroscopy. *Angewandte Chemie International Edition*.

[17] Madine J., Jack E., Stockley P. G., Radford S. E., Serpell L. C., Middleton D. A. (2008). Structural insights into the polymorphism of amyloid-like fibrils formed by region 20-29 of amylin revealed by solid-state NMR and X-ray fiber diffraction. *Journal of the American Chemical Society*.

[18] Luca S., Yau W.-M., Leapman R., Tycko R. (2007). Peptide conformation and supramolecular organization in amylin fibrils: constraints from solid-state NMR. *Biochemistry*.

[19] Jayasinghe S. A., Langen R. (2004). Identifying structural features of fibrillar islet amyloid polypeptide using site-directed spin labeling. *The Journal of Biological Chemistry*.

[20] Apostolidou M., Jayasinghe S., Langen R. (2005). Structure of membrane-bound IAPP studied by EPR spectroscopy. *Biophysical Journal*.

[21] Ling Y. L., Strasfeld D. B., Shim S.-H., Raleigh D. P., Zanni M. T. (2009). Two-dimensional infrared spectroscopy provides evidence of an intermediate in the membrane-catalyzed assembly of diabetic amyloid. *The Journal of Physical Chemistry B*.

[22] Shim S.-H., Gupta R., Ling Y. L., Strasfeld D. B., Raleigh D. P., Zanni M. T. (2009). Two-dimensional IR spectroscopy and isotope labeling defines the pathway of amyloid formation with residue-specific resolution. *Proceedings of the National Academy of Sciences of the United States of America*.

[23] Shim S.-H., Strasfeld D. B., Ling Y. L., Zanni M. T. (2007). Automated 2D IR spectroscopy using a mid-IR pulse shaper and application of this technology to the human islet amyloid polypeptide. *Proceedings of the National Academy of Sciences of the United States of America*.

[24] Sarroukh R., Goormaghtigh E., Ruysschaert J.-M., Raussens V. (2013). ATR-FTIR: A ‘rejuvenated’ tool to investigate amyloid proteins. *Biochimica et Biophysica Acta—Biomembranes*.

[25] Jaikaran E. T. A. S., Higham C. E., Serpell L. C. (2001). Identification of a novel human islet amyloid polypeptide *β*-sheet domain and factors influencing fibrillogenesis. *Journal of Molecular Biology*.

[26] Moriarty D. F., Raleigh D. P. (1999). Effects of sequential proline substitutions on amyloid formation by human amylin_20-29_. *Biochemistry*.

[27] Lau T.-L., Ambroggio E. E., Tew D. J. (2006). Amyloid-*β* peptide disruption of lipid membranes and the effect of metal ions. *Journal of Molecular Biology*.

[28] Lee J. C., Langen R., Hummel P. A., Gray H. B., Winkler J. R. (2004). *α*-synuclein structures from fluorescence energy-transfer kinetics: implications for the role of the protein in Parkinson's disease. *Proceedings of the National Academy of Sciences of the United States of America*.

[29] Profit A. A., Felsen V., Chinwong J., Mojica E.-R. E., Desamero R. Z. B. (2013). Evidence of *π*-stacking interactions in the self-assembly of hIAPP22-29. *Proteins: Structure, Function and Bioinformatics*.

[30] Li S., Micic M., Orbulescu J., Whyte J. D., Leblanc R. M. (2012). Human islet amyloid polypeptide at the air-aqueous interface: a Langmuir monolayer approach. *Journal of the Royal Society Interface*.

[31] Lopes D. H. J., Meister A., Gohlke A., Hauser A., Blume A., Winter R. (2007). Mechanism of islet amyloid polypeptide fibrillation at lipid interfaces studied by infrared reflection absorption spectroscopy. *Biophysical Journal*.

[32] Yan E. C., Wang Z., Fu L. (2015). Proteins at interfaces probed by chiral vibrational sum frequency generation spectroscopy. *The Journal of Physical Chemistry B*.

[33] Yan E. C. Y., Fu L., Wang Z., Liu W. (2014). Biological macromolecules at interfaces probed by chiral vibrational sum frequency generation spectroscopy. *Chemical Reviews*.

[34] Shen Y. R. (1989). Surface properties probed by second-harmonic and sum-frequency generation. *Nature*.

[35] Eisenthal K. B. (1996). Liquid interfaces probed by second-harmonic and sum-frequency spectroscopy. *Chemical Reviews*.

[36] Richmond G. L. (2002). Molecular bonding and interactions at aqueous surfaces as probed by vibrational sum frequency spectroscopy. *Chemical Reviews*.

[37] Shultz M. J., Schnitzer C., Simonelli D., Baldelli S. (2000). Sum frequency generation spectroscopy of the aqueous interface: ionic and soluble molecular solutions. *International Reviews in Physical Chemistry*.

[38] Chen Z., Shen Y. R., Somorjai G. A. (2002). Studies of polymer surfaces by sum frequency generation vibrational spectroscopy. *Annual Review of Physical Chemistry*.

[39] Wang J., Clarke M. L., Chen X., Even M. A., Johnson W. C., Chen Z. (2005). Molecular studies on protein conformations at polymer/liquid interfaces using sum frequency generation vibrational spectroscopy. *Surface Science*.

[40] Su X. C., Cremer P. S., Shen Y. R., Somorjai G. A. (1997). High-pressure CO oxidation on Pt(111) monitored with infrared-visible sum frequency generation (SFG). *Journal of the American Chemical Society*.

[41] Zhuang X., Miranda P. B., Kim D., Shen Y. R. (1999). Mapping molecular orientation and conformation at interfaces by surface nonlinear optics. *Physical Review B—Condensed Matter and Materials Physics*.

[42] Jubb A. M., Hua W., Allen H. C. (2012). Environmental chemistry at vapor/water interfaces: insights from vibrational sum frequency generation spectroscopy. *Annual review of physical chemistry*.

[43] Stokes G. Y., Chen E. H., Buchbinder A. M., Paxton W. F., Keeley A., Geiger F. M. (2009). Atmospheric heterogeneous stereochemistry. *Journal of the American Chemical Society*.

[44] Martinez I. S., Peterson M. D., Ebben C. J. (2011). On molecular chirality within naturally occurring secondary organic aerosol particles from the central Amazon Basin. *Physical Chemistry Chemical Physics*.

[45] Chen X. Y., Clarke M. L., Wang J., Chen Z. (2005). Sum frequency generation vibrational spectroscopy studies on molecular conformation and orientation of biological molecules at interfaces. *International Journal of Modern Physics B*.

[46] Chen X., Wang J., Kristalyn C. B., Chen Z. (2007). Real-time structural investigation of a lipid bilayer during its interaction with melittin using sum frequency generation vibrational spectroscopy. *Biophysical Journal*.

[47] Wang J., Chen X. Y., Clarke M. L., Chen Z. (2005). Detection of chiral sum frequency generation vibrational spectra of proteins and peptides at interfaces in situ. *Proceedings of the National Academy of Sciences of the United States of America*.

[48] Stokes G. Y., Gibbs-Davis J. M., Boman F. C. (2007). Making ‘sense’ of DNA. *Journal of the American Chemical Society*.

[49] Walter S. R., Geiger F. M. (2010). DNA on stage: showcasing oligonucleotides at surfaces and interfaces with second harmonic and vibrational sum frequency generation. *Journal of Physical Chemistry Letters*.

[50] Liu J., Conboy J. C. (2004). Direct measurement of the transbilayer movement of phospholipids by sum-frequency vibrational spectroscopy. *Journal of the American Chemical Society*.

[51] Liu J., Conboy J. C. (2005). Structure of a gel phase lipid bilayer prepared by the Langmuir-Blodgett/ Langmuir-Schaefer method characterized by sum-frequency vibrational spectroscopy. *Langmuir*.

[52] Anglin T. C., Liu J., Conboy J. C. (2007). Facile lipid flip-flop in a phospholipid bilayer induced by gramicidin A measured by sum-frequency vibrational spectroscopy. *Biophysical Journal*.

[53] Tian C. S., Shen Y. R. (2014). Recent progress on sum-frequency spectroscopy. *Surface Science Reports*.

[54] Qiao L., Ge A., Osawa M., Ye S. (2013). Structure and stability studies of mixed monolayers of saturated and unsaturated phospholipids under low-level ozone. *Physical Chemistry Chemical Physics*.

[55] Wang Z., Fu L., Yan E. C. Y. (2013). C–H stretch for probing kinetics of self-assembly into macromolecular chiral structures at interfaces by chiral sum frequency generation spectroscopy. *Langmuir*.

[56] Meister K., Strazdaite S., DeVries A. L. (2014). Observation of ice-like water layers at an aqueous protein surface. *Proceedings of the National Academy of Sciences of the United States of America*.

[57] Chen X., Chen Z. (2006). SFG studies on interactions between antimicrobial peptides and supported lipid bilayers. *Biochimica et Biophysica Acta (BBA)—Biomembranes*.

[58] Rao Y., Kwok S. J. J., Lombardi J., Turro N. J., Eisenthal K. B. (2014). Label-free probe of HIV-1 TAT peptide binding to mimetic membranes. *Proceedings of the National Academy of Sciences of the United States of America*.

[59] Li Z. G., Weeraman C. N., Azam M. S., Osman E., Gibbs-Davis J. M. (2015). The thermal reorganization of DNA immobilized at the silica/buffer interface: a vibrational sum frequency generation investigation. *Physical Chemistry Chemical Physics*.

[60] Wurpel G. W. H., Sovago M., Bonn M. (2007). Sensitive probing of DNA binding to a cationic lipid monolayer. *Journal of the American Chemical Society*.

[61] Fu L., Liu J., Yan E. C. Y. (2011). Chiral sum frequency generation spectroscopy for characterizing protein secondary structures at interfaces. *Journal of the American Chemical Society*.

[62] Fu L., Ma G., Yan E. C. Y. (2010). In situ misfolding of human islet amyloid polypeptide at interfaces probed by vibrational sum frequency generation. *Journal of the American Chemical Society*.

[63] Rzeźnicka I. I., Pandey R., Schleeger M., Bonn M., Weidner T. (2014). Formation of lysozyme oligomers at model cell membranes monitored with sum frequency generation spectroscopy. *Langmuir*.

[64] Engel M. F. M., Vandenakker C. C., Schleeger M., Velikov K. P., Koenderink G. H., Bonn M. (2012). The polyphenol EGCG inhibits amyloid formation less efficiently at phospholipid interfaces than in bulk solution. *Journal of the American Chemical Society*.

[65] Xiao D., Fu L., Liu J., Batista V. S., Yan E. C. Y. (2012). Amphiphilic adsorption of human islet amyloid polypeptide aggregates to lipid/aqueous interfaces. *Journal of Molecular Biology*.

[66] Lambert A. G., Davies P. B., Neivandt D. J. (2005). Implementing the theory of sum frequency generation vibrational spectroscopy: a tutorial review. *Applied Spectroscopy Reviews*.

[67] Wang H.-F., Gan W., Lu R., Rao Y., Wu B.-H. (2005). Quantitative spectral and orientational analysis in surface sum frequency generation vibrational spectroscopy (SFG-VS). *International Reviews in Physical Chemistry*.

[68] Shen Y.-R. (1984). *The Principles of Nonlinear Optics*.

[69] Boyed R. W. (2003). *Nonlinear Optics*.

[70] Wang H.-F., Velarde L., Gan W., Fu L. (2015). Quantitative sum-frequency generation vibrational spectroscopy of molecular surfaces and interfaces: lineshape, polarization, and orientation. *Annual Review of Physical Chemistry*.

[71] Lin C. K., Yang L., Hayashi M. (2014). Theory and applications of sum-frequency generations. *Journal of the Chinese Chemical Society*.

[72] Belkin M. A., Shen Y. R. (2005). Non-linear optical spectroscopy as a novel probe for molecular chirality. *International Reviews in Physical Chemistry*.

[73] Simpson G. J. (2004). Molecular origins of the remarkable chiral sensitivity of second-order nonlinear optic. *ChemPhysChem*.

[74] Haupert L. M., Simpson G. J. (2009). Chirality in nonlinear optics. *Annual Review of Physical Chemistry*.

[75] Moad A. J., Simpson G. J. (2004). A unified treatment of selection rules and symmetry relations for sum-frequency and second harmonic spectroscopies. *Journal of Physical Chemistry B*.

[76] Wang J., Clarke M. L., Chen Z. (2004). Polarization mapping: a method to improve sum frequency generation spectral analysis. *Analytical Chemistry*.

[77] Hebda J. A., Saraogi I., Magzoub M., Hamilton A. D., Miranker A. D. (2009). A peptidomimetic approach to targeting pre-amyloidogenic states in type II diabetes. *Chemistry and Biology*.

[78] Hore D. K., King J. L., Moore F. G., Alavi D. S., Hamamoto M. Y., Richmond G. L. (2004). Ti : Sapphire-based picosecond visible-infrared sum-frequency spectroscopy from 900–3100 cm^−1^. *Applied Spectroscopy*.

[79] Hommel E. L., Ma G., Allen H. C. (2001). Broadband vibrational sum frequency generation spectroscopy of a liquid surface. *Analytical Sciences*.

[80] Smith J. P., Hinson-Smith V. (2004). Product review: SFG coming of age. *Analytical Chemistry*.

[81] Ma G., Allen H. C. (2003). Surface studies of aqueous methanol solutions by vibrational broad bandwidth sum frequency generation spectroscopy. *Journal of Physical Chemistry B*.

[82] Fu L., Xiao D. Q., Wang Z. G., Batista V. S., Yan E. C. Y. (2013). Chiral sum frequency generation for in situ probing proton exchange in antiparallel beta-sheets at interfaces. *Journal of the American Chemical Society*.

[83] Relini A., Marano N., Gliozzi A. (2014). Probing the interplay between amyloidogenic proteins and membranes using lipid monolayers and bilayers. *Advances in Colloid and Interface Science*.

[84] Braak H., Braak E. (1991). Neuropathological stageing of Alzheimer-related changes. *Acta Neuropathologica*.

[85] Chiti F., Dobson C. M. (2006). Protein misfolding, functional amyloid, and human disease. *Annual Review of Biochemistry*.

[86] Barth A., Zscherp C. (2002). What vibrations tell us about proteins. *Quarterly Reviews of Biophysics*.

[87] Tamm L. K., Tatulian S. A. (1997). Infrared spectroscopy of proteins and peptides in lipid bilayers. *Quarterly Reviews of Biophysics*.

[88] Fu L., Wang Z., Yan E. C. Y. (2011). Chiral vibrational structures of proteins at interfaces probed by sum frequency generation spectroscopy. *International Journal of Molecular Sciences*.

[89] Saraogi I., Hebda J. A., Becerril J., Estroff L. A., Miranker A. D., Hamilton A. D. (2010). Synthetic *α*-helix mimetics as agonists and antagonists of islet amyloid polypeptide aggregation. *Angewandte Chemie*.

[90] Ehrnhoefer D. E., Bieschke J., Boeddrich A. (2008). EGCG redirects amyloidogenic polypeptides into unstructured, off-pathway oligomers. *Nature Structural and Molecular Biology*.

[91] Meng F., Abedini A., Plesner A., Verchere C. B., Raleigh D. P. (2010). The Flavanol (−)-epigallocatechin 3-gallate inhibits amyloid formation by islet amyloid polypeptide, disaggregates amyloid fibrils, and protects cultured cells against IAPP-induced toxicity. *Biochemistry*.

[92] Dupuis N. F., Wu C., Shea J.-E., Bowers M. T. (2011). The amyloid formation mechanism in human IAPP: dimers have *β*-strand monomer-monomer interfaces. *Journal of the American Chemical Society*.

[93] Morriss-Andrews A., Shea J.-E. (2015). Computational studies of protein aggregation: methods and applications. *Annual Review of Physical Chemistry*.

[94] Bellesia G., Shea J.-E. (2009). Effect of Β-sheet propensity on peptide aggregation. *The Journal of Chemical Physics*.

[95] Li H., Ye S., Wei F., Ma S., Luo Y. (2012). In situ molecular-level insights into the interfacial structure changes of membrane-associated prion protein fragment [118–135] investigated by sum frequency generation vibrational spectroscopy. *Langmuir*.

[96] Jiang Z., de Messieres M., Lee J. C. (2013). Membrane remodeling by *α*-synuclein and effects on amyloid formation. *Journal of the American Chemical Society*.

[97] Li S., Leblanc R. M. (2014). Aggregation of insulin at the interface. *The Journal of Physical Chemistry B*.

[98] Mifflin A. L., Velarde L., Ho J. (2015). Accurate line shapes from sub-1 cm^−1^ resolution sum frequency generation vibrational spectroscopy of *α*-pinene at room temperature. *The Journal of Physical Chemistry A*.

[99] Velarde L., Zhang X.-Y., Lu Z., Joly A. G., Wang Z., Wang H.-F. (2011). Communication: spectroscopic phase and lineshapes in high-resolution broadband sum frequency vibrational spectroscopy: resolving interfacial inhomogeneities of ‘identical’ molecular groups. *Journal of Chemical Physics*.

[100] Velarde L., Wang H.-F. (2013). Unified treatment and measurement of the spectral resolution and temporal effects in frequency-resolved sum-frequency generation vibrational spectroscopy (SFG-VS). *Physical Chemistry Chemical Physics*.

[101] Fu L., Zhang Y., Wei Z. H., Wang H. F. (2014). Intrinsic chirality and prochirality at air/R-(+)- and S-(−)-limonene interfaces: spectral signatures with interference chiral sum-frequency generation vibrational spectroscopy. *Chirality*.

[102] Yamaguchi S., Tahara T. (2008). Heterodyne-detected electronic sum frequency generation: ‘up’ versus ‘down’ alignment of interfacial molecules. *Journal of Chemical Physics*.

[103] Stiopkin I. V., Jayathilake H. D., Bordenyuk A. N., Benderskii A. V. (2008). Heterodyne-detected vibrational sum frequency generation spectroscopy. *Journal of the American Chemical Society*.

[104] Hu D., Yang Z., Chou K. C. (2013). Interactions of polyelectrolytes with water and ions at air/water interfaces studied by phase-sensitive sum frequency generation vibrational spectroscopy. *The Journal of Physical Chemistry C*.

[105] Hua W., Jubb A. M., Allen H. C. (2011). Electric field reversal of Na_2_SO_4_, (NH 4)_2_SO_4_, and Na_2_CO_3_ relative to CaCl_2_ and NaCl at the air/aqueous interface revealed by heterodyne detected phase-sensitive sum frequency. *Journal of Physical Chemistry Letters*.

[106] Anfuso C. L., Snoeberger R. C., Ricks A. M. (2011). Covalent attachment of a rhenium bipyridyl CO_2_ reduction catalyst to rutile TiO_2_. *Journal of the American Chemical Society*.

[107] Anfuso C. L., Xiao D., Ricks A. M., Negre C. F. A., Batista V. S., Lian T. (2012). Orientation of a series of CO_2_ reduction catalysts on single crystal TiO_2_ probed by phase-sensitive vibrational sum frequency generation spectroscopy (PS-VSFG). *Journal of Physical Chemistry C*.

[108] Xiong W., Laaser J. E., Mehlenbacher R. D., Zanni M. T. (2011). Adding a dimension to the infrared spectra of interfaces using heterodyne detected 2D sumfrequency generation (HD 2D SFG) spectroscopy. *Proceedings of the National Academy of Sciences of the United States of America*.

[109] Laaser J. E., Zanni M. T. (2013). Extracting structural information from the polarization dependence of one- and two-dimensional sum frequency generation spectra. *The Journal of Physical Chemistry A*.

[110] Laaser J. E., Skoff D. R., Ho J.-J. (2014). Two-dimensional sum-frequency generation reveals structure and dynamics of a surface-bound peptide. *Journal of the American Chemical Society*.

[111] Fujisaki H., Straub J. E. (2005). Vibrational energy relaxation in proteins. *Proceedings of the National Academy of Sciences of the United States of America*.

[112] Fujisaki H., Yagi K., Hirao K., Straub J. E. (2007). Quantum dynamics of N-methylacetamide studied by the vibrational configuration interaction method. *Chemical Physics Letters*.

